# Grandmother Speaks of the Old Country

**DOI:** 10.3201/eid1208.AD1208

**Published:** 2006-08

**Authors:** Lola Haskins

**Keywords:** Keywords: Grandmother, Lola Haskins, Another Dimension, germs, poetry, poem

That year there were many deaths in the village.
Germs flew like angels from one house to the next
and every family gave up its own. Mothers
died at their mending. Children fell at school.
Of three hundred twenty, there were eleven left.
Then, quietly, the sun set on a day when no one
died. And the angels whispered among themselves.
And that evening, as he sat on the stone steps,
your grandfather felt a small wind on his neck
when all the trees were still. And he would tell us
always, how he had felt that night, on the skin
of his own neck, the angels, passing.

Copyright 2004 by Lola Haskins. Reprinted from Desire Lines: New and Selected Poems, BOA Editions, 2004, by permission of the author and the publisher through American Life in Poetry, an initiative of Ted Kooser, the 2004-2006 poet laureate consultant in poetry to the Library of Congress; the American Life in Poetry project is supported by The Poetry Foundation, The Library of Congress, and the Department of English at the University of Nebraska-Lincoln.

